# Clinical case of animal hoarding – characterization and management of a new disorder

**DOI:** 10.1192/j.eurpsy.2021.1272

**Published:** 2021-08-13

**Authors:** L. Lopes, A. Certo, S. Pereira, Â. Venâncio

**Affiliations:** Department Of Psychiatry And Mental Health, Centro Hospitalar de Vila Nova de Gaia e Espinho, Vila Nova de Gaia, Portugal

**Keywords:** Animal hoarding disorder, psychopathology, schizophrénia, Comorbid symptoms

## Abstract

**Introduction:**

Animal hoarding is characterized by hoarding of a large number of animals without providing minimum conditions of nutrition and sanitation, accompanied by lack of insight for the behavior and by social isolation. Despite studies detecting an increasing incidence, the behavior is still poorly understood.

**Objectives:**

To review clinical evidence on animal hoarding and to report a clinical case.

**Methods:**

We report a clinical case based on patient’s history and clinical data, along with a review of the literature on animal hoarding. The terms “Noah syndrome” and “animal hoarding disorder” were searched on PubMed® database.

**Results:**

We present the case of a 51-years-old woman, living alone, with higher education. His first contact with psychiatry was in August 2019 upon aggravated self-neglect and behavioral disorganization. She was living with around 40 cats, her home was extremely deteriorated. In December 2019 she was admitted to a psychiatric unit. A schizophrenia diagnosis was established and pharmacological treatment was initiated. She was discharged to a chronic psychiatric institution. Studies found out that animal hoarders are typically middle age/older women living alone in squalid conditions. Animal hoarding is characterized by a chronic course and intense emotional attachment to animals. It seems to be associated with traumatic situations, as well as mental disorders such as schizophrenia or dementia. Published data on intervention and treatment is still limited.

**Conclusions:**

Animal hoarding phenomenon requires further investigation, regarding developmental risk factors and co-morbid mental disorders. Comprehensive approaches to clinical intervention and management strategies in animal hoarding are necessary.

